# Supplementary material for: Kidney cancer PDOXs reveal patient‐specific pro‐malignant effects of antiangiogenics and its molecular traits
Source: EMBO Mol Med. 2020 Nov 5;12(12):e11889. doi: 10.15252/emmm.201911889 (PMC7721359; doi:10.15252/emmm.201911889)
Supplement: Supplementary file 1 — Appendix [file EMMM-12-e11889-s001.pdf]

APPENDIX - TABLE OF CONTENT

Description of each Appendix Table and Figure……………………………………………………..…….Page 1

Appendix Table S1. Patient and tumor characteristics of all implanted specimens and engrafted Ren-PDOXs…………………………………………………………………………………………..……Page 2

Appendix Table S2. Complete list of patients and tumor characteristics of paired implanted specimen and engrafted Ren-PDOX…………………………………………………………Page 4

Appendix Table S3. Haloplex custom panel…………………………………………………………….Page 6

Appendix Table S4. SNVs/indels in Ren-PDXs………………………………………………………….Page 7

Appendix Figure S1. GSEA analysis of TCGA……………………………………………………………Page 9

Appendix Table S5. Actual N and P values of each experiment………………………………Page 10

Appendix Table S1. Patient and tumor characteristics of all implanted specimens and engrafted Ren-PDOXs.

Table includes patient demographics and tumor characteristics of all implanted tumors and engrafted Ren-PDOXs (n/a, not available or assessed). In A, the percentage is calculated over the total number of implanted specimens. In B, the percentage is calculated as engraftment over the number of implanted specimens for each group.

Appendix Table S2. Complete list of patients and tumor characteristics of paired implanted specimen and engrafted Ren-PDOX.

Table provides patient demographics, tumor characteristics at implantation (Furhman grade, TNM classification), assessment of Ren-PDOX generation (+, positive or -, negative engraftment) and spontaneous lung metastasis in mice (+, present or -, absent). Ren46 derived from a recurrence after nephrectomy, all others specimens were obtained from primary untreated clear cell renal cell carcinoma. Ren-PDOX name derived from paired Specimen ID. n/a, not available or assessed. In TNM staging, where not specified, pN and/or pM status correspond to pN0 and pM0. *The patient developed metachronous metastasis.

Appendix Table S3. Haloplex custom panel

List of genes included in Haloplex custom panel analyzed by targeted-NGS sequencing.

Appendix Table S4. SNVs/indels in Ren-PDXs identified by high-throughput sequencing.

List of SNVs and indels found in genes included in Haloplex panel by targeted-NGS or WES. Only non-synonymous/stop gain SNVs and frameshift indels novel or with MAF$\leq$0.01 are reported. ID, identity of variants as reported in public database (rs in Genome Browser, COSM in COSMIC, VARSOME, MU in ICGC Data Portal). FATHMM prediction was provided by COSMIC database.

Appendix Figure S1.

A) Representative GSEA enrichment plots for gene sets enriched in Ren13 and in Ren50 tumors.
B) Fold change and p-value for the overlapping 19 genes in TCGA analysis.

Appendix Table S1. Patient and tumor characteristics of all implanted specimens and engrafted Ren-PDOXs.

A-All implanted	n	implanted (%)
**Total**	56	
TUMOR CHARACTERISTICS		
**TNM**	**55/56**	
pT status		
**pT1**	17/55	30.9
**pT2**	7/55	12.7
**pT3**	27/55	49.1
**pT4**	4/55	7.3
pN status		
**pNx**	6/55	10.9
**pN0**	45/55	81.8
**pN1**	4/55	7.3
pM status		-
**pMx**	0/55	0.0
**pM0**	45/55	81.8
**pM1**	10/55	18.2
**metachronous**	n/a	-
**Fuhrman grade**	**56/56**	
1	3/56	5.4
2	15/56	26.8
3	24/56	42.9
4	14/56	25.0

B-Engrafted Ren-PDOX	n	engrafted (%)
PATIENT CHARACTERISTICS		
**Total**	27	
Female	14	
Male	13	
**Age**	**years**	
range	24-89	
median	64	
TUMOR CHARACTERISTICS		
**TNM**	**26/27**	
pT status		

**pT1**	5/17	29.4
**pT2**	6/7	85.7
**pT3**	13/27	48.1
**pT4**	2/4	50.0
pN status		
**pNx**	2/6	33.3
**pN0**	21/45	46.7
**pN1**	3/4	75.0
pM status		
**pMx**	0	0,0
**pM0**	17/45	37.8
**pM1**	9/10	90.0
**metachronous**	6	n/a
**Fuhrman grade**	**27/27**	
**1**	1/3	33.3
**2**	8/15	53.3
**3**	10/24	41.7
**4**	8/14	57.1

Appendix Table S2. Complete list of patients and tumor characteristics of paired implanted specimen and engrafted Ren-PDOX.

	PATIENT		TUMOR		Ren-PDOX	
Specimen ID	Sex	Age	Fuhrman Grade	TNM staging	Ren-PDOX generation	Ren-PDOX lung metastasis
**Ren1**	n/a	n/a	3	pT3b	-	n/a
**Ren7**	F	52	3	pT3b	+	-
**Ren8**	n/a	n/a	2	pT1b	-	n/a
**Ren13***	F	39	3	pT2	+	+
**Ren15**	n/a	n/a	4	pT1b	-	n/a
**Ren17**	n/a	n/a	2	pT1b	-	n/a
**Ren18***	M	80	3	pT2	+	-
**Ren20**	n/a	n/a	3	pT2	-	n/a
**Ren23**	n/a	n/a	2	pT3b	-	n/a
**Ren26**	n/a	n/a	2	pT1a	-	n/a
**Ren28***	F	78	4	pT3b pN1	+	+
**Ren30**	n/a	n/a	2	pT1b	-	n/a
**Ren31**	n/a	n/a	2	pT3b	-	n/a
**Ren33**	n/a	n/a	3	pT3b	-	n/a
**Ren36**	M	75	2	pT1a	+	-
**Ren38**	M	55	2	pT3a pN1	+	+
**Ren41**	F	77	2	pT3a pM1	+	+
**Ren42**	n/a	n/a	3	pT3a	-	n/a
**Ren45**	n/a	n/a	3	pT1a	-	n/a
**Ren46**	F	58	3	n/a	+	-
**Ren47**	F	89	3	pT3a	+	+
**Ren48**	n/a	n/a	1	pT1a	-	n/a
**Ren50**	M	75	4	pT3a pN1 pM1	+	+
**Ren57**	n/a	n/a	2	pT1b	-	n/a
**Ren58***	F	50	3	pT3a	+	-
**Ren59**	M	73	2	pT2b	+	-
**Ren60**	n/a	n/a	4	pT3a	-	n/a
**Ren61**	n/a	n/a	3	pT1b	-	n/a
**Ren62**	n/a	n/a	1	pT1b	-	n/a
**Ren64**	F	64	3	pT2a	+	-
**Ren65***	M	40	4	pT3b	+	-
**Ren66**	n/a	n/a	4	pT1b	-	n/a
**Ren67**	F	66	3	pT2b pM1	+	n/a

**Ren68**	M	64	2	pT1a	**+**	-
**Ren70**	F	78	2	pT1a	**+**	-
**Ren71**	F	62	1	pT2b	**+**	-
**Ren75**	n/a	n/a	3	pT3a	-	n/a
**Ren86**	F	70	3	pT1a<br>pM1	**+**	**+**
**Ren87***	M	57	3	pT3a	**+**	-
**Ren88**	n/a	n/a	3	pT1a	-	n/a
**Ren91**	n/a	n/a	3	pT3a<br>pN1	-	n/a
**Ren95**	M	55	4	pT3c<br>pM1	**+**	-
**Ren96**	M	64	4	pT3b<br>pN0<br>pM1	**+**	**+**
**Ren98**	M	69	4	pT3a<br>pNx<br>pM1	**+**	**+**
**Ren101**	F	41	4	pT4<br>pN0<br>pM1	**+**	-
**Ren102**	M	24	2	pT3a<br>pNx	**+**	-
**Ren103**	M	65	4	pT4<br>pN0<br>pM1	-	n/a
**Ren107**	M	48	4	pT3c	-	n/a
**Ren108**	M	53	4	pT4	**+**	**+**
**Ren110**	F	65	3	pT3b<br>pNx	-	n/a
**Ren111**	F	62	2	pT1<br>pN0<br>pM1	**+**	-
**Ren112**	M	56	3	pT3a<br>pNx	-	n/a
**Ren113**	M	50	4	pT3c<br>pNx	-	n/a
**Ren115**	F	56	3	pT3c	-	n/a
**Ren116**	M	81	3	pT3b	-	n/a
**Ren118**	M	67	3	pT4a<br>pNx	-	n/a

Appendix Table S3. Haloplex custom panel

Gene									
ABCA12	BRIP1	DKK2	FANCA	HRAS	KDM8	MYB	PIK3CB	RET	SYNE1
ABCA7	BUB1B	DKK3	FANCC	HSP90AA1	KDR	MYC	PIK3CD	RICTOR	TBK1
ABL1	CARD11	DKK4	FANCD2	IDH1	KEAP1	MYCL1	PIK3CG	RNF43	TCERG1
ABL2	CASP8	DMD	FANCE	IDH2	KIT	MYCN	PIK3R1	RPTOR	TCF7L2
ACVR1B	CBL	DNM2	FANCF	IGF1R	KLF6	MYD88	PIK3R2	RUNX1	TEK
ACVR2A	CBLB	DNMT1	FANCG	IGFBP7	KLHDC4	MYO1B	PIK3R3	SBDS	TERT
ADAMTS20	CBLC	DNMT3A	FAS	IKBKE	KRAS	NALCN	PIKFYVE	SCN5A	TET1
AFF2	CCND1	DNMT3B	FBN1	IKZF1	LDHA	NBN	PIM1	SDHB	TET2
AKT1	CCNE1	DOCK2	FBN2	IL7R	LEFTY1	NCOA2	PIP4K2A	SDHC	TET3
AKT2	CD79B	DPP6	FBXO11	IMPA1	LGR6	NF1	PIP4K2B	SDHD	TGFBR1
AKT3	CDC27	ECT2L	FBXW7	IMPAD1	LRP5	NF2	PKM2	SERPINA9	TGFBR2
ALK	CDC42EP2	EDNRB	FGFR1	INPP1	MAGI2	NFE2L2	PLK2	SERPINB1	TMPRSS2
ALOX12B	CDC73	EGFR	FGFR2	INPP4A	MAP2K1	NFKB1	PLK3	SERPINB2	TNFAIP3
APC	CDH1	EIF4EBP1	FGFR3	INPP4B	MAP2K2	NFKB2	PMS1	SERPINB3	TNFRSF14
AR	CDH10	EP300	FGFR4	INPP5B	MAP2K4	NKX2-1	PMS2	SERPINB4	TOP1
ARAF	CDK12	EPC1	FH	INPP5D	MAP3K5	NOTCH1	PNRC1	SERPINB5	TP53
ARHGAP26	CDK4	EPHA3	FLCN	INSR	MAP3K8	NOTCH2	POLE	SERPINE1	TP63
ARID1A	CDK5	EPHA5	FLT1	IRS1	MAP3K9	NOTCH3	PPIP5K2	SERPINI1	TSC1
ARID1B	CDK6	EPHA6	FLT3	IRS2	MAP7	NOTCH4	PPP2R1A	SERPINI2	TSC2
ARID2	CDK8	EPHA7	FMN2	ISYNA1	MAPK12	NPM1	PRDM1	SETD2	TSHR
ASXL1	CDKN2A	EPHA8	FOXL2	ITPKA	MCL1	NRAS	PREX2	SF3B1	U2AF1
ATM	CDKN2B	EPHB1	FUBP1	ITPKB	MDM2	NSD1	PRKAR1A	SHQ1	VHL
ATP6V0D2	CDKN2C	EPHB4	FZD3	JAK1	MDM4	NTRK1	PRKCI	SKI	WAS
ATR	CEBPA	EPHB6	GATA1	JAK2	MEN1	NTRK2	PTCH1	SKIL	WBSCR17
ATRX	CHEK1	ERBB2	GATA2	JAK3	MET	NTRK3	PTEN	SLC16A4	WHSC1
AURKA	CHEK2	ERBB3	GATA3	JUN	MIER3	OR10R2	PTPN11	SLC9A9	WHSC1L1
AXIN1	CIC	ERBB4	GNA11	KAT6A	MINPP1	PAK7	PTPN12	SMAD2	WRN
AXIN2	CREBBP	ERCC2	GNAQ	KAT6B	MITF	PALB2	PTPRD	SMAD3	WT1
BAI3	CRKL	ERCC3	GNAS	KDM1A	MLH1	PARK2	PTPRS	SMAD4	XIRP2
BAP1	CRLF2	ERCC4	GOLPH3	KDM2A	MLH3	PARP1	PXDN	SMAD7	XPA
BARD1	CSF1R	ERCC5	GPC3	KDM2B	MLL	PAX5	RAD51	SMARCA4	XPC
BCL2L1	CSMD1	ERG	GPC6	KDM3B	MLL2	PBRM1	RAF1	SMARCB1	XPO1
BCL6	CTNNA2	ESR1	GRIK3	KDM4A	MLL3	PCDH15	RARA	SMO	YAP1
BCOR	CTNNB1	ETV1	GRIN2A	KDM4B	MLST8	PDGFRA	RASA1	SOCS1	YES1
BIRC2	CYLD	ETV6	GSK3B	KDM4C	MPL	PDGFRB	RASA2	SOX2	ZIM2
BLM	DAXX	EXT1	H3F3A	KDM5A	MSH2	PDZRN3	RASA3	SPOP	ZNRF3
BMPR1A	DDR2	EXT2	HDAC2	KDM5B	MSH3	PHF6	RASA4	SRC	ZRSR2
BRAF	DICER1	EZH2	HIF1A	KDM5C	MSH6	PHOX2B	RB1	SRSF2	
BRCA1	DIS3	FAM123B	HMGA2	KDM6A	MTOR	PIK3C2G	RECQL4	STK11	
BRCA2	DKK1	FAM46C	HNF1A	KDM6B	MUTYH	PIK3CA	REL	SUFU	

Appendix Table S4. SNVs/indels in Ren-PDXs identified by high-throughput sequencing.

GENE	NM Ref	Ren13	Ren28	Ren38	Ren50	Ren86	Affected protein	ID	FATHMM prediction
**ARID1A**	NM_006015.4		c.268A>G				*Ser90Gly*	rs752026201 # COSM6389743 # MU90572047	Likely Benign
			c.676G>C				*Ala226Pro*	rs746698854	Likely Benign
			c.680C>A				*Pro227Gln*	rs1334915537 # COSM6035010	Likely Benign
			c.776_778delCCT				*Ser265del*	novel	Uncertain Significance
**ATM**	NM_000051.3			c.8510delA			*Lys2838Asnfs*19*	novel	Pathogenic
				c.4388T>G			*Phe1463Cys*	rs138327406	Benign
**BAP1**	NM_004656.2	c.2110delG					*Val704Serfs*32*	COSM1732149 # MU121559453	Pathogenic
			c.610delG				*Asp204Thrfs*27*	novel	Pathogenic
**ERBB3**	NM_001982.2					c.3727C>A	*Leu1243Ile*	novel	Uncertain Significance
**IGF1R**	NM_000875.3		c.173A>T			c.173A>T	*Tyr58Phe*	novel	Likely Benign
**KIT**	NM_000222.2	c.2554G>A					*Val852Ile*	rs555650901 # COSM1734911	Uncertain Significance
**MLL3**	NM_170606.2		c.750G>A				*Trp250**	novel	Pathogenic
				c.3791T>C			*Val1264Ala*	rs1190930208	Likely Benign
					c.8390delA		*Lys2797Argfs*26*	rs747256476 # COSM328978 # MU4523379	Uncertain Significance
**MTOR**	NM_004958.3	c.7280T>G					*Leu2427Arg*	COSM2119114 # MU29857217	Likely Pathogenic
**PBRM1**	NM_018313.4	c.3697G>T					*Glu1233**	novel	Pathogenic
						c.1051_1053delTATinsA	*Tyr351Argfs*5*	novel	Pathogenic
					c.667delA		*Ile223**	novel	Pathogenic

**SETD2**	NM_014159.6			c.843_844insA			*Glu282Argfs*9*	COSM4613100	Likely Pathogenic
**TET2**	NM_001127208.1			c.5642A>G			*His1881Arg*	rs1417392445 # COSM43445	Uncertain Significance
**TSC1**	NM_000368.3					c.2959G>T	*Glu987**	novel	Pathogenic
**TSC2**	NM_000548.3	c.2873A>G					*Asn958Ser*	rs397515104 # COSM6986393	Likely Benign
**VHL**	NM_000551.2	c.262T>C					*Trp88Arg*	COSM17953 # MU113499507	Pathogenic
			c.240T>A				*Ser80Arg*	COSM14290 # MU113497180	Pathogenic
				c.343C>A			*His115Asn*	COSM17752 # MU625781	Likely Pathogenic

Appendix Figure S1.

A)

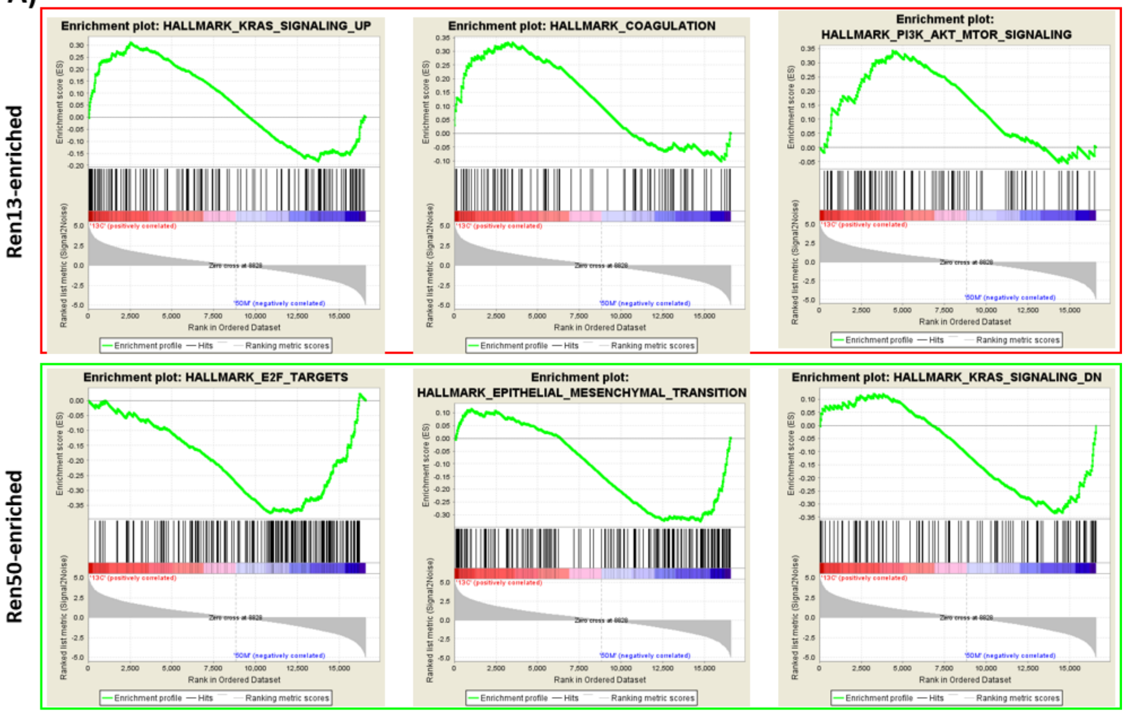

B)

	effect_M	pvalue_M	effect_T	pvalue_T	effect_S	pvalue_S
ADAM8	0,70	0,000	0,64	0,000	0,66	0,000
AKR1B10	0,97	0,008	0,62	0,023	0,73	0,007
ALDH1A3	0,59	0,001	0,43	0,001	0,43	0,001
CAPN9	0,40	0,044	0,31	0,028	0,38	0,007
CBR4	-0,19	0,003	-0,21	0,000	-0,23	0,000
CBX8	0,19	0,015	0,12	0,040	0,14	0,011
CCNA1	1,19	0,000	0,99	0,000	1,01	0,000
CFB	0,53	0,002	0,49	0,000	0,58	0,000
CYP39A1	-0,86	0,000	-0,64	0,000	-0,76	0,000
DUSP6	-0,29	0,003	-0,17	0,019	-0,21	0,003
GPR19	0,49	0,000	0,46	0,000	0,47	0,000
MAGIX	-0,29	0,024	-0,24	0,010	-0,25	0,007
MAP7	-0,61	0,000	-0,63	0,00	-0,64	0,00
MEFV	0,48	0,001	0,46	0,00	0,41	0,00
MFSD6	-0,29	0,001	-0,25	0,00	-0,30	0,00
MMP9	0,52	0,042	1,06	0,00	0,97	0,00
SATB1	-0,39	0,000	-0,25	0,00	-0,34	0,00
THRB	-0,61	0,000	-0,45	0,00	-0,58	0,00
TSPAN7	-0,97	0,000	-0,78	0,00	-0,84	0,00

Appendix Table S5. Actual N and P values of each experiment

Figure #	Experiment/Group	N values	Statistics P values	
Fig. 1B		C:7; DC:7; Bv:7	DC vs C: NS-NS-0.0169-0.0373	BEVA vs C: NS-0.0175-0.0385-0.0390
Fig. 1C		C:7; DC:7; Bv:7	DC vs C: 0.0134	BEVA vs C: 0.0233
Fig. 1D		C:7; DC:7; Bv:7	DC vs C: 0.0022	BEVA vs C: 0.0065
Fig. 1E		C:7; DC:7; Bv:7	DC vs C: 0.0087	BEVA vs C: 0.0087
Fig. 1F		C:7; DC:7; Bv:7	DC vs C: 0.0004	BEVA vs C: 0.001
Fig. 1G		C:7; DC:7; Bv:7	DC vs C: 0.0262	BEVA vs C: 0.0262
Fig. 1H	Mets. Incidence	C:14; DC:13; Bv:5	DC vs C: 0.0341	BEVA vs C: 0.0198
	Number of foci	C:14; DC:13; Bv:5	DC vs C: 0.0364	BEVA vs C: 0.0152
Fig. 2A		n=26	NS	
Fig. 3A	Ren13	C:11; DC:11; Bv:11	DC vs C: NS-NS-0.0014-0.0009-0.0071-0.0115-0.014	BEVA vs C: NS-0.0093-0.0009-0.0077-NS-0.0222-NS
	Ren86	C:9; DC:9; Bv:6	DC vs C: NS	BEVA vs C: NS
	Ren28	C:7; DC:6; Bv:5	DC vs C: 0.0476-0.0079-0.0079-0.0079-0.0079-0.0079	BEVA vs C: 0.0159-0.0079-0.0079-0.0079-0.0079-0.0079
	Ren50	C:13; DC:10; Bv:6	DC vs C: 0.0490-0.0099-0.0058-0.0058-0.0087	BEVA vs C: NS-0.0041-0.0023-0.0035-0.0065
Fig. 3B	Ren13	C:11; DC:10; Bv:13	DC vs C: 0.0016	BEVA vs C: 0.0054
	Ren86	C:7; DC:9; Bv:5	DC vs C: 0.0033	BEVA vs C: 0.0325
	Ren28	C:7; DC:6; Bv:5	DC vs C: 0.0079	BEVA vs C: 0.0079
	Ren50	C:13; DC:10; Bv:6	DC vs C: 0.0286	BEVA vs C: 0.0035
Fig. 3C	Ren13	C:5; DC:4; Bv:5	DC vs C: 0.0159	BEVA vs C: 0.0079
	Ren86	C:5; DC:5; Bv:5	DC vs C: 0.0079	BEVA vs C: 0.0079
	Ren28	C:4; DC:4; Bv:5	DC vs C: 0.0079	BEVA vs C: 0.0079
	Ren50	C:7; DC:7; Bv:6	DC vs C: 0.0012	BEVA vs C: 0.0017
Fig. 3D	Ren13	C:5; DC:4; Bv:5	DC vs C: 0.0317	BEVA vs C: 0.0159
	Ren86	C:5; DC:5; Bv:5	DC vs C: 0.0048	BEVA vs C: 0.0095
	Ren28	C:4; DC:4; Bv:5	DC vs C: 0.0286	BEVA vs C: 0.0159
	Ren50	C:7; DC:7; Bv:6	DC vs C: 0.0167	BEVA vs C: 0.0333
Fig. 3E	Ren13	C:9; DC:11; Bv:11	DC vs C: NS	BEVA vs C: NS
	Ren86	C:9; DC:9; Bv:4	DC vs C: NS	BEVA vs C: NS
	Ren28	C:7; DC:6; Bv:5	DC vs C: 0.0241	BEVA vs C: 0.0127
	Ren50	C:13; DC:10; Bv:6	DC vs C: 0.0031	BEVA vs C: 0.0174
Fig. 4B	Ren13	C:7; DC:6; Bv:8	DC vs C: 0.0083	BEVA vs C: 0.0034
	Ren86	C:19; DC:10; Bv:5	DC vs C: 0.048	BEVA vs C: 0.0063
	Ren28	C:5; DC:5; Bv:6	DC vs C: NS 0.4902	BEVA vs C: NS 0.819
	Ren50	C:5; DC:6; Bv:6	DC vs C: NS 0.7401	BEVA vs C: NS 0.1368
Fig. 4C	Ren13	C:11; DC:9; Bv:8	DC vs C: 0.0053	BEVA vs C: 0.0065
	Ren86	C:11; DC:9; Bv:7	DC vs C: 0.0034	BEVA vs C: 0.015
	Ren28	C:10; DC:12; Bv:6	DC vs C: NS 0.0831	BEVA vs C: NS >0.9999
	Ren50	C:8; DC:7; Bv:9	DC vs C: NS 0.244	BEVA vs C: NS 0.9822
Fig. 4D	Ren13BM	n=14	Capsular Invasion: 0.0363	Tumor Invasion: 0.0083
	Ren28	n=13	Capsular Invasion: NS	Tumor Invasion: NS
Fig. 5B	Ren13BM	C:13; DC:5; Bv:7	DC vs C: 0.0351	BEVA vs C: NS
	Ren28	C:8; DC:7; Bv:5	DC vs C: NS 0.8327	BEVA vs C: NS 0.5060
Fig. 5C	Ren13BM	C:13; DC:5; Bv:7	DC vs C: 0.0308	BEVA vs C: NS
	Ren28	C:8; DC:7; Bv:5	DC vs C: NS >0.9999	BEVA vs C: NS 0.7063
Fig. 6D	MAP7 MVI	n=39	Yes vs No: 0.0463	
	MAP7 RV	n=40	Yes vs No: 0.0061	
	MAP7 INVASION	n=41	Yes vs No: RV 0.0317	
	ALDH1A3 MVI	n=42	Yes vs No: 0.0017	
	ALDH1A3 RV	n=43	Yes vs No: 0.0148	
	ALDH1A3 INVASION	n=44	Yes vs No: RV 0.0032	
Fig. 7B		Ren13: 5	Ren13 vs Ren28: 0.0159	
		Ren28: 5	Ren13 vs Ren50: 0.0043	
		Ren13: 6	Ren28 vs Ren86: 0.0159	
		Ren13: 4	Ren50 vs Ren86: 0.0095	
Fig. 7C		n=7	Yes vs No: 0.0082	
Fig. 7D		n=7	p: 0.0095	
Fig. 7F		n=15	Chi-square test for independence p=0.0157	Chi-square test for trend p=0.030
Fig. 7G		n=15	p: 0.008	
