# Supplementary material for: Kidney cancer PDOXs reveal patient‐specific pro‐malignant effects of antiangiogenics and its molecular traits
Source: EMBO Mol Med. 2020 Nov 5;12(12):e11889. doi: 10.15252/emmm.201911889 (PMC7721359; doi:10.15252/emmm.201911889)
Supplement: Supplementary file 2 — Expanded View Figures PDF [file EMMM-12-e11889-s002.pdf]

Expanded View Figures

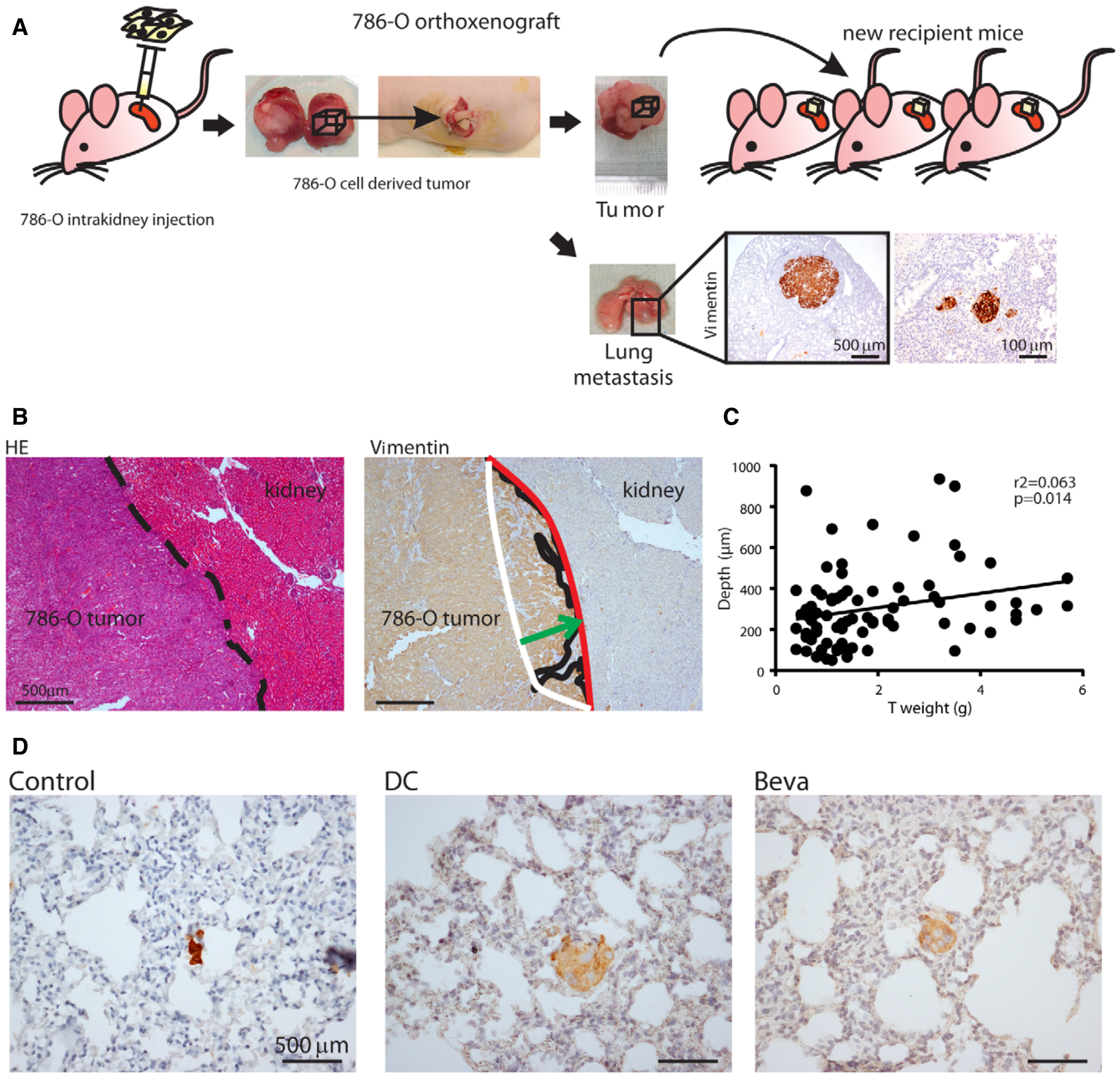

Figure EV1. Generation of 786-O orthoxenograft and evaluation of anti-VEGF/R treatment effects.

A Representation of protocol for the establishment of 786-O orthoxenograft. First 786-O tumor was generated from cell injected into renal capsule. 786-O orthoxenografts were then established and perpetuated by implantation of pieces from 786-O tumor into kidney of new recipient mice. A representative image of 786-O tumor is shown. Lung macrometastases, visible at sacrifice on lung surface and confirmed by vimentin staining in FFPE section and micrometastases in vimentin-stained section are shown (representative IHC images, 4X and 20X respectively).

B Representative images of tumor-kidney interface (black lines) of 786-O tumor in HE and vimentin-stained sections (4X). Red and white lines define, respectively, the front and the rear edge of tumor protrusion into kidney. Green arrow perpendicularly connecting red and white lines was used to measure depth of invasion.

C Correlation between depth (Depth, µm) and weight (T weight, g) evaluated in untreated tumors (n = 81 samples; $r^2 = 0.063$, $P < 0.05$ by Spearman test).

D Representative images of lung metastasis stained by vimentin in control and treated 786-O tumors.

 © 2020 The Authors Published under the terms of the CC BY 4.0 license

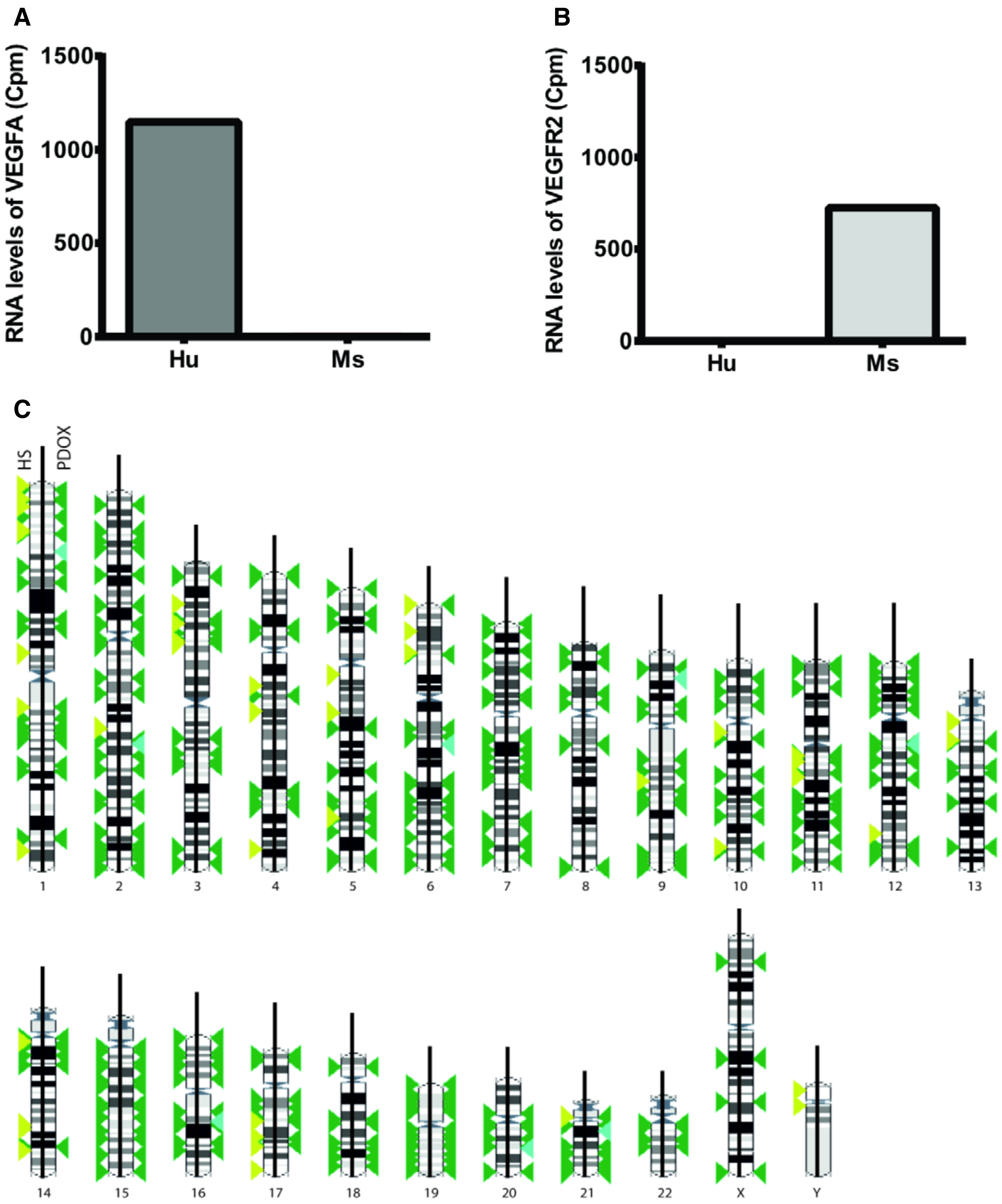

Figure EV2. Extended molecular characterization of Ren-PDOX.

A, B Species-specific RNA sequencing analysis of Human and Mouse VEGF-A and VEGFR2.

C Distribution of variants in human biopsy and Ren-PDOX. In karyotype, distribution of variants called in human specimen (HS) and Ren-PDOX (PDOX), respectively, on left and right site of each chromosome are color coded for common (green), human-only (yellow), or PDOX-only (blue).

© 2020 The Authors Published under the terms of the CC BY 4.0 license

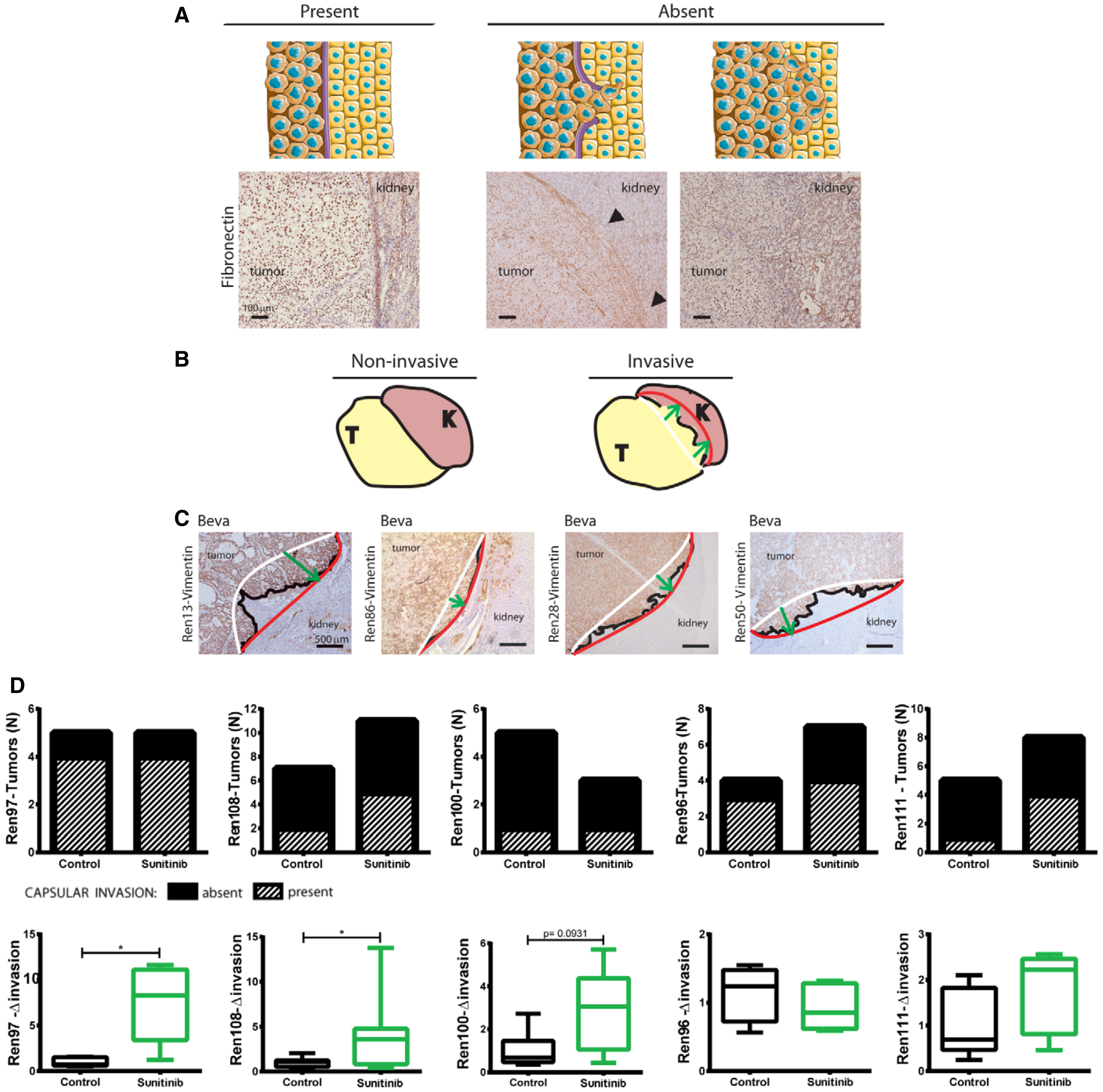

Figure EV3. Capsular invasion and invasive front of Ren-PDOXs.

A Schematic illustration and representative images of FN stained sections (10X) showing different grades of capsular invasion (CI). Capsular invasion is scored as present or absent depending on tumor (T) infiltration inside normal kidney parenchyma (K) at tumor-kidney interface. Arrows indicate point of completely invaded capsule.

B, C Schematic illustration and representative images of invasive front in vimentin-stained sections of Beva-treated Ren13, Ren86, Ren28, and Ren50-PDOX tumors are shown (4X). Front (red line) and rear (white line) edges of tumor protrusion into kidney determine the depth of invasion (green arrow) perpendicularly connecting red and white lines. Their correspondent quantifications of fold-invasion are shown in Fig 4C.

D Effects of sunitinib treatment on an independent series of five new Ren-PDOXs showing variable therapy-induced effects on capsular invasion (top, bar graphs) and effects on tumor front invasion (bottom, box plots). 5–10 animals per tumor were used. Box plots represent median, Q1/Q3 and max/min value whiskers analyzed by Mann–Whitney test *$P < 0.05$.

 © 2020 The Authors Published under the terms of the CC BY 4.0 license

Figure EV4. Validation of TCGA gene candidates.

Gene expression of each candidate gene from TCGA analysis in the invasion-annotated series of 39 patients (GSE29609). MVI, microvascular invasion; and RV, renal vein involvement. Box plots represent median, Q1/Q3 and max/min value whiskers.

▶

© 2020 The Authors Published under the terms of the CC BY 4.0 license

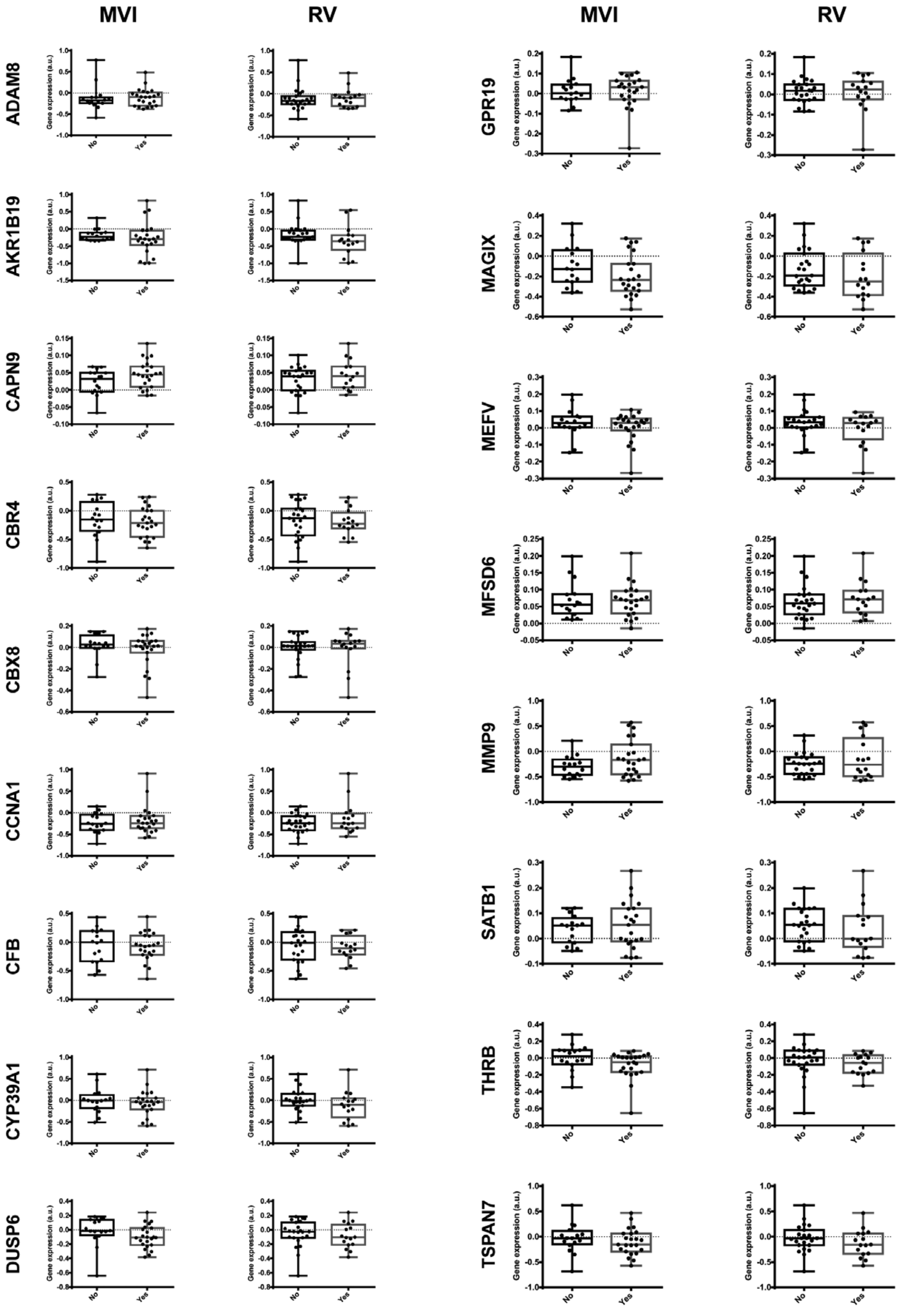

Figure EV4.

 © 2020 The Authors Published under the terms of the CC BY 4.0 license

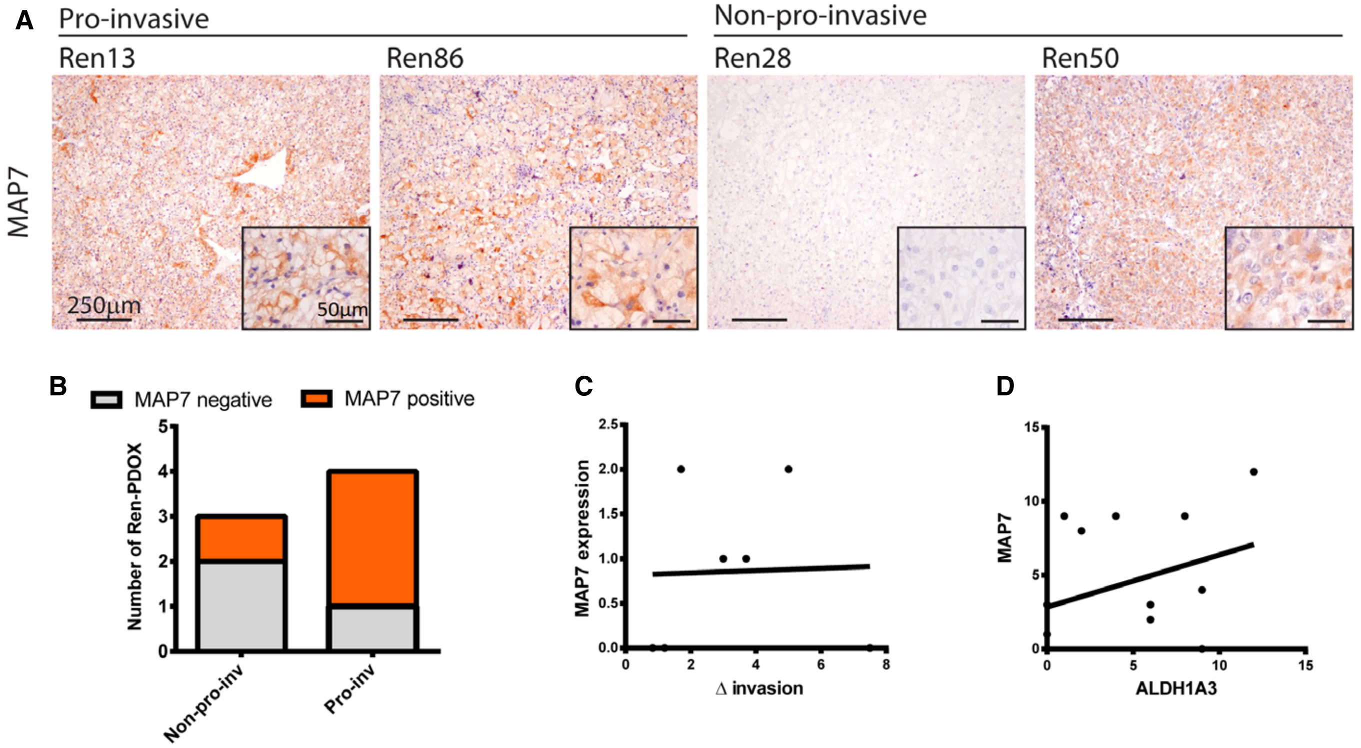

Figure EV5. MAP7 is not associated nor predicts pro-invasiveness.

A Representative images of MAP7 expression on pro-invasive (Ren13 and Ren86) and non-pro-invasive (Ren28 and Ren50) tumors.

B Representation of MAP7 expression on pro-invasive and non-pro-invasive Ren-PDOXs after sunitinib treatment (Chi-square test $P = 0.07$).

C, D Lack of correlation of pre-treatment MAP7 protein levels by IHC with increased tumor invasion found after sunitinib treatment (C) and with ALDH1A3 protein levels by IHC (D). $n = 7$, Spearman's non-parametric correlation, P = ns.

© 2020 The Authors Published under the terms of the CC BY 4.0 license
