# Supplementary figures and images for: Kidney cancer PDOXs reveal patient‐specific pro‐malignant effects of antiangiogenics and its molecular traits
Source: EMBO Mol Med. 2020 Nov 5;12(12):e11889. doi: 10.15252/emmm.201911889 (PMC7721359; doi:10.15252/emmm.201911889)

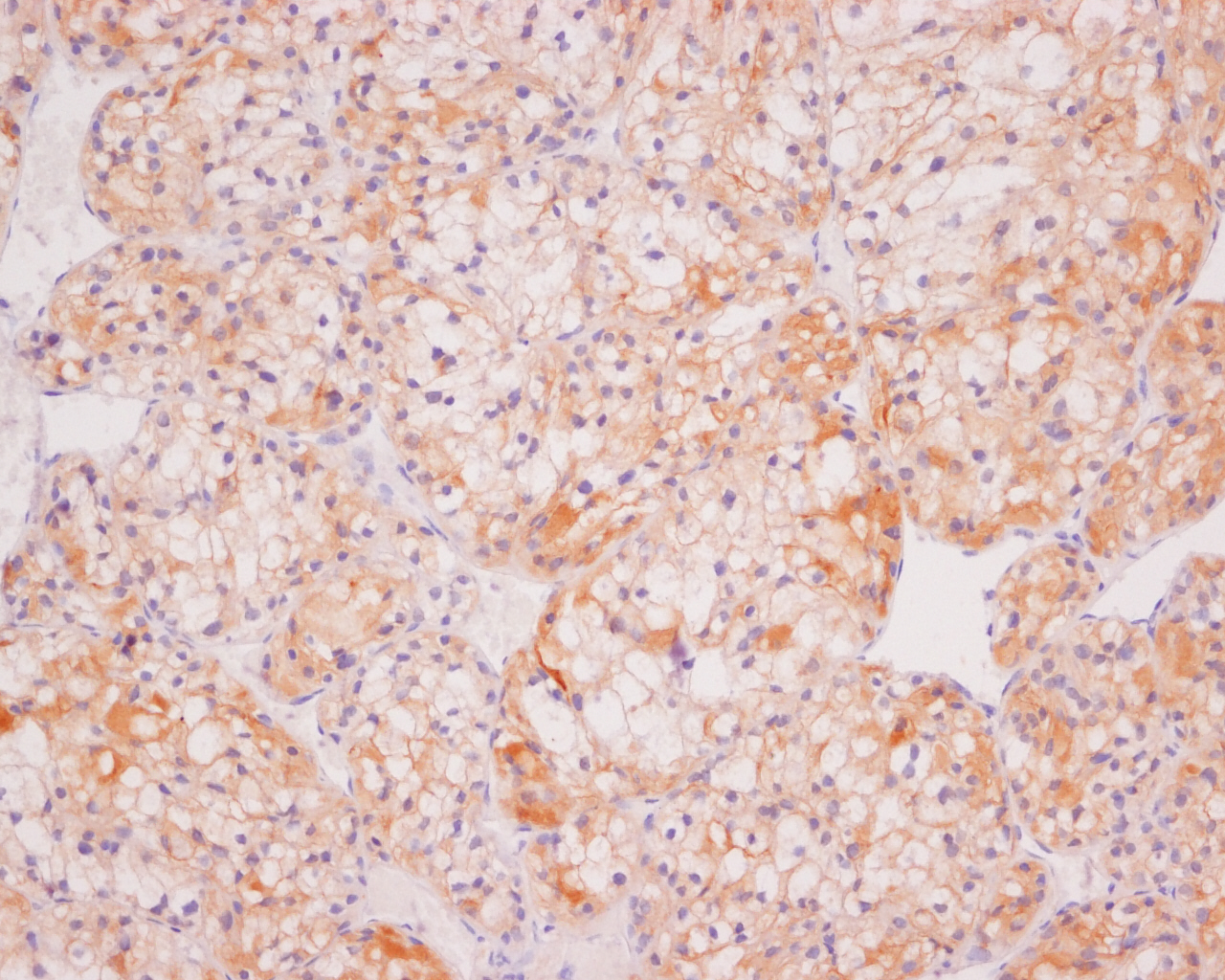

Supplement: Supplementary file 3 — Source Data for Expanded View/Appendix [file EMMM-12-e11889-s005.zip › EMM-11889-EV-source-data/Fig.EV5A_Ren13_inset.jpg]

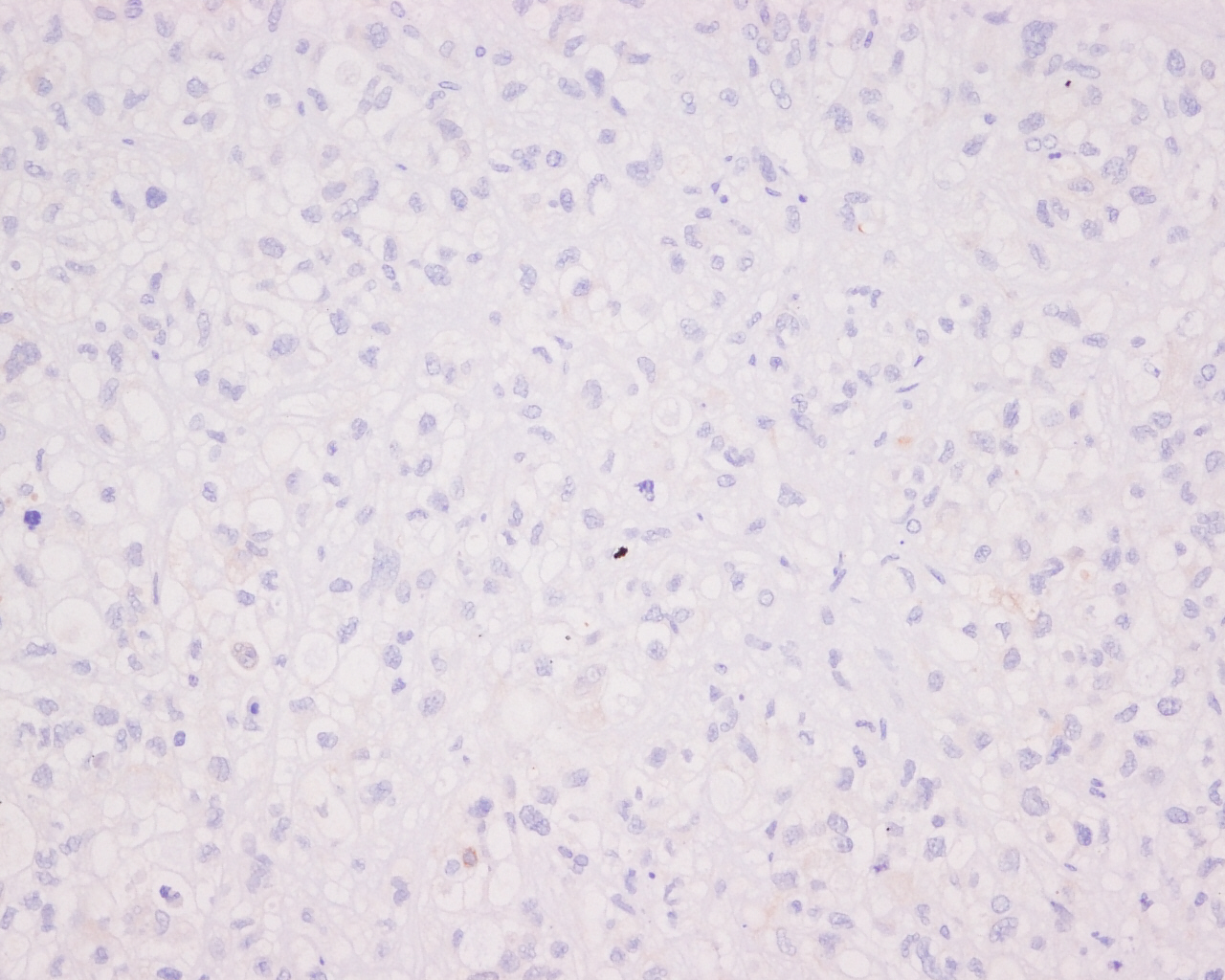

Supplement: Supplementary file 3 — Source Data for Expanded View/Appendix [file EMMM-12-e11889-s005.zip › EMM-11889-EV-source-data/Fig.EV5A_Ren28_inset.jpg]

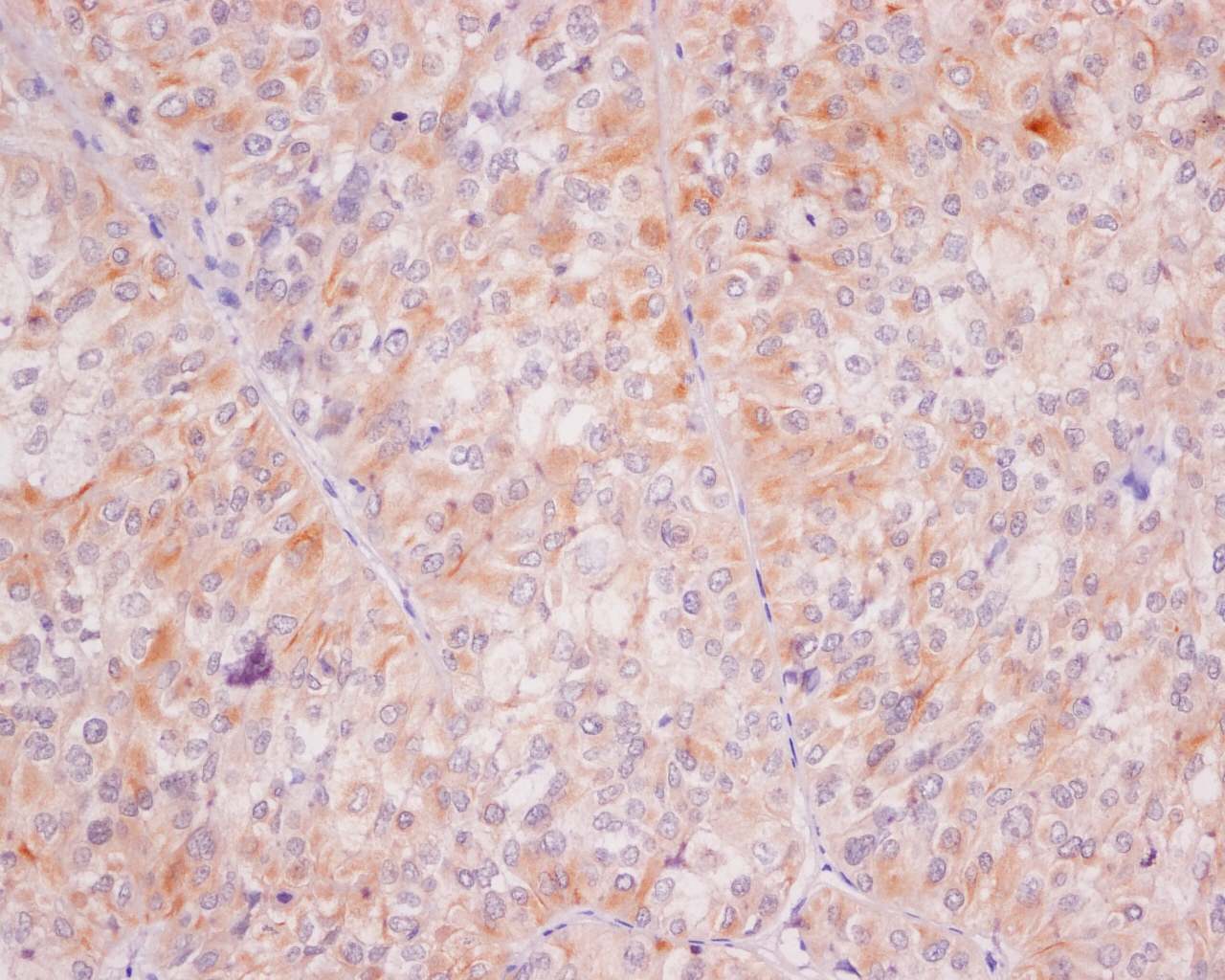

Supplement: Supplementary file 3 — Source Data for Expanded View/Appendix [file EMMM-12-e11889-s005.zip › EMM-11889-EV-source-data/Fig.EV5A_Ren50_inset.jpg]

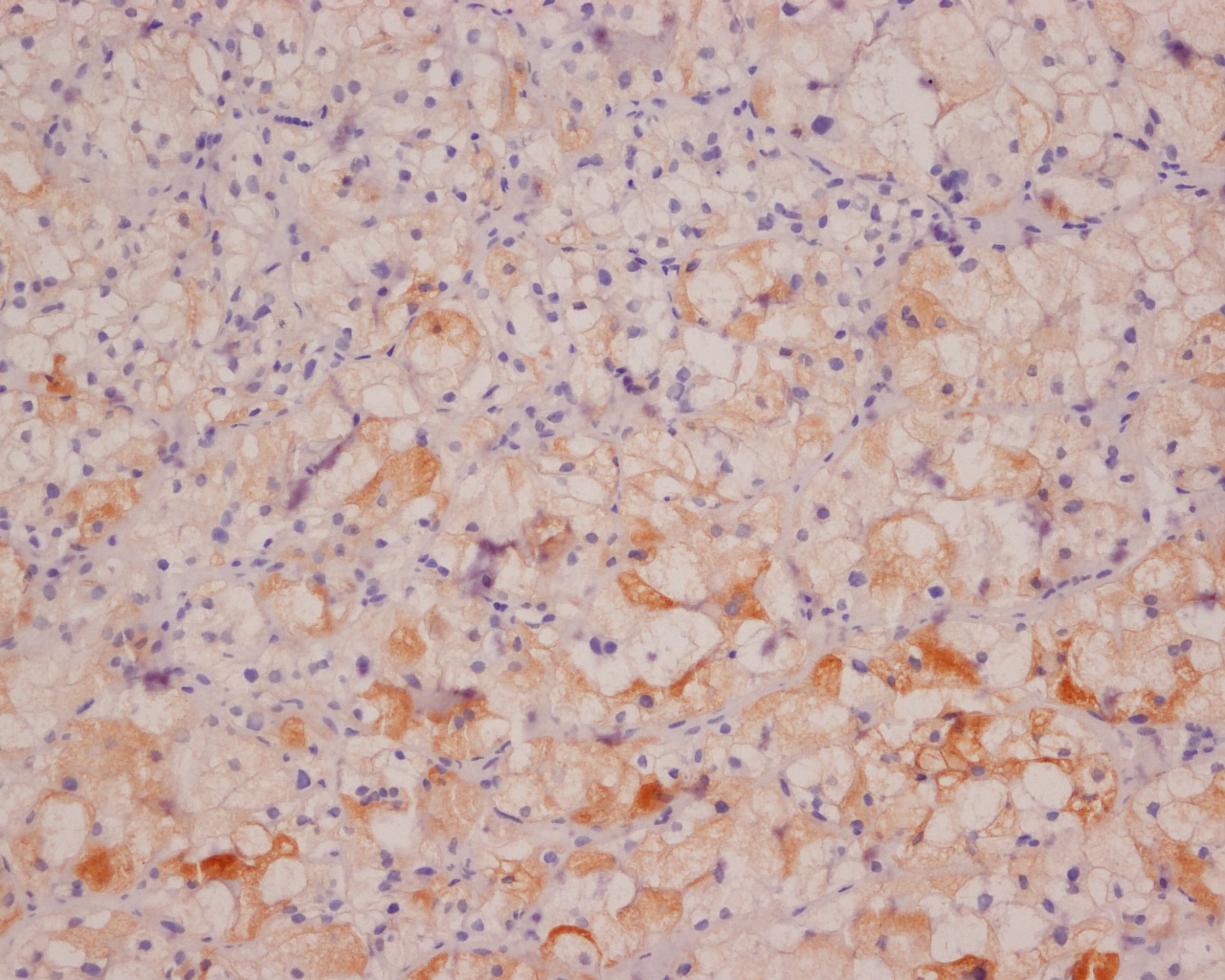

Supplement: Supplementary file 3 — Source Data for Expanded View/Appendix [file EMMM-12-e11889-s005.zip › EMM-11889-EV-source-data/Fig.EV5A_Ren86_inset.jpg]

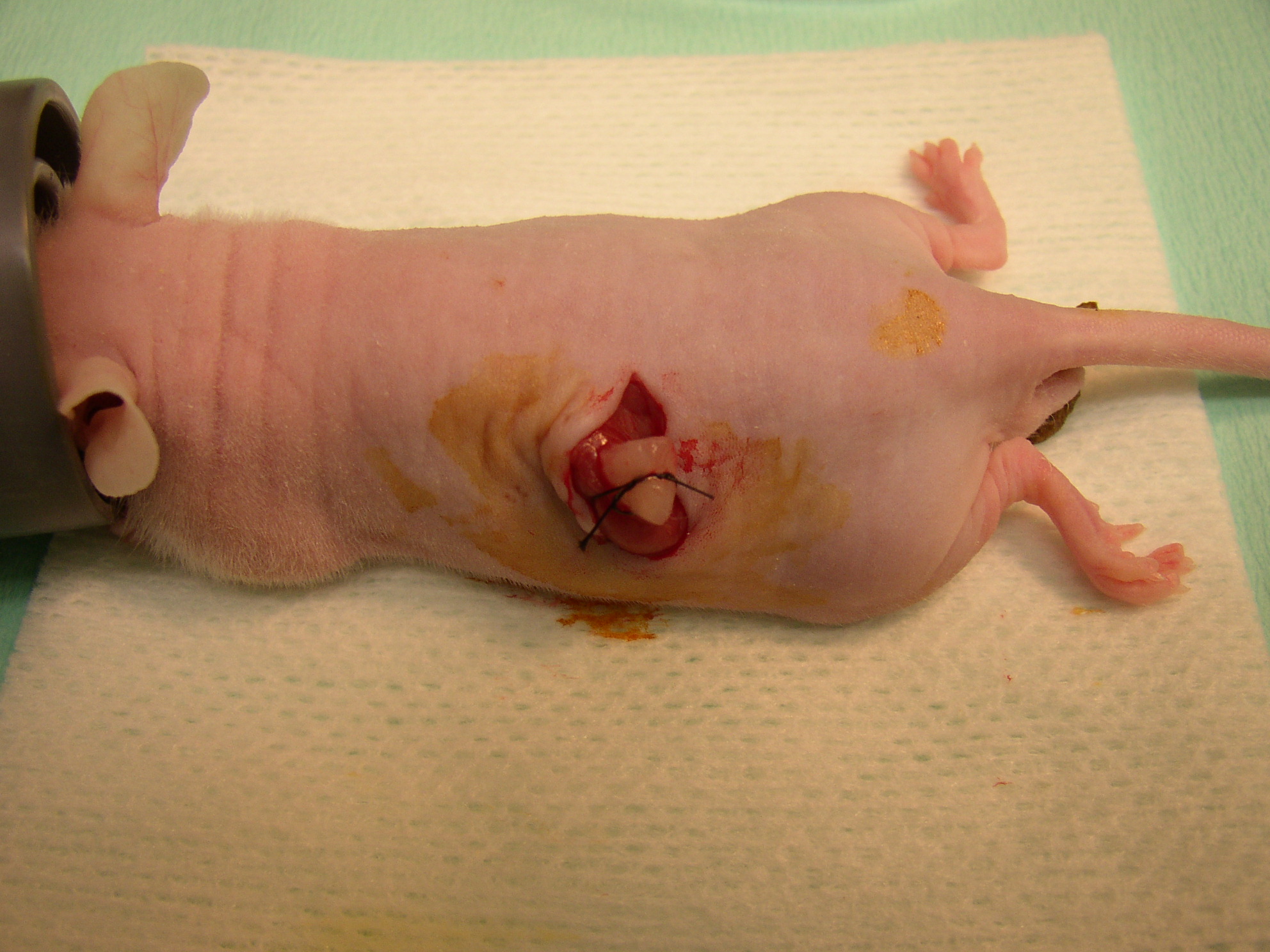

Supplement: Supplementary file 5 — Source Data for Figure 2 [file EMMM-12-e11889-s003.jpg]

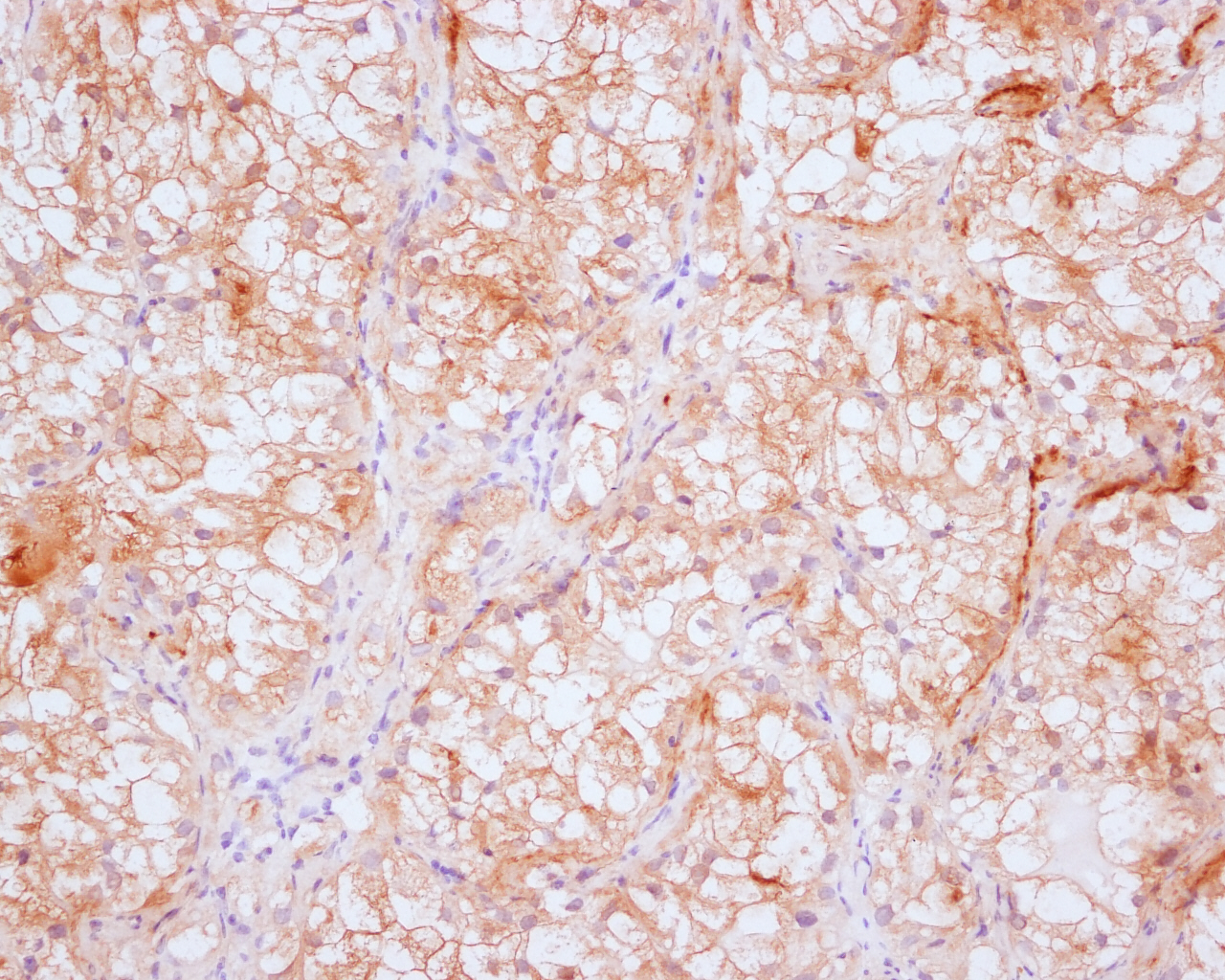

Supplement: Supplementary file 6 — Source Data for Figure 7 [file EMMM-12-e11889-s004.zip › EMM-11889-Fig7-source-data/Fig.7A_Ren13_inset.jpg]

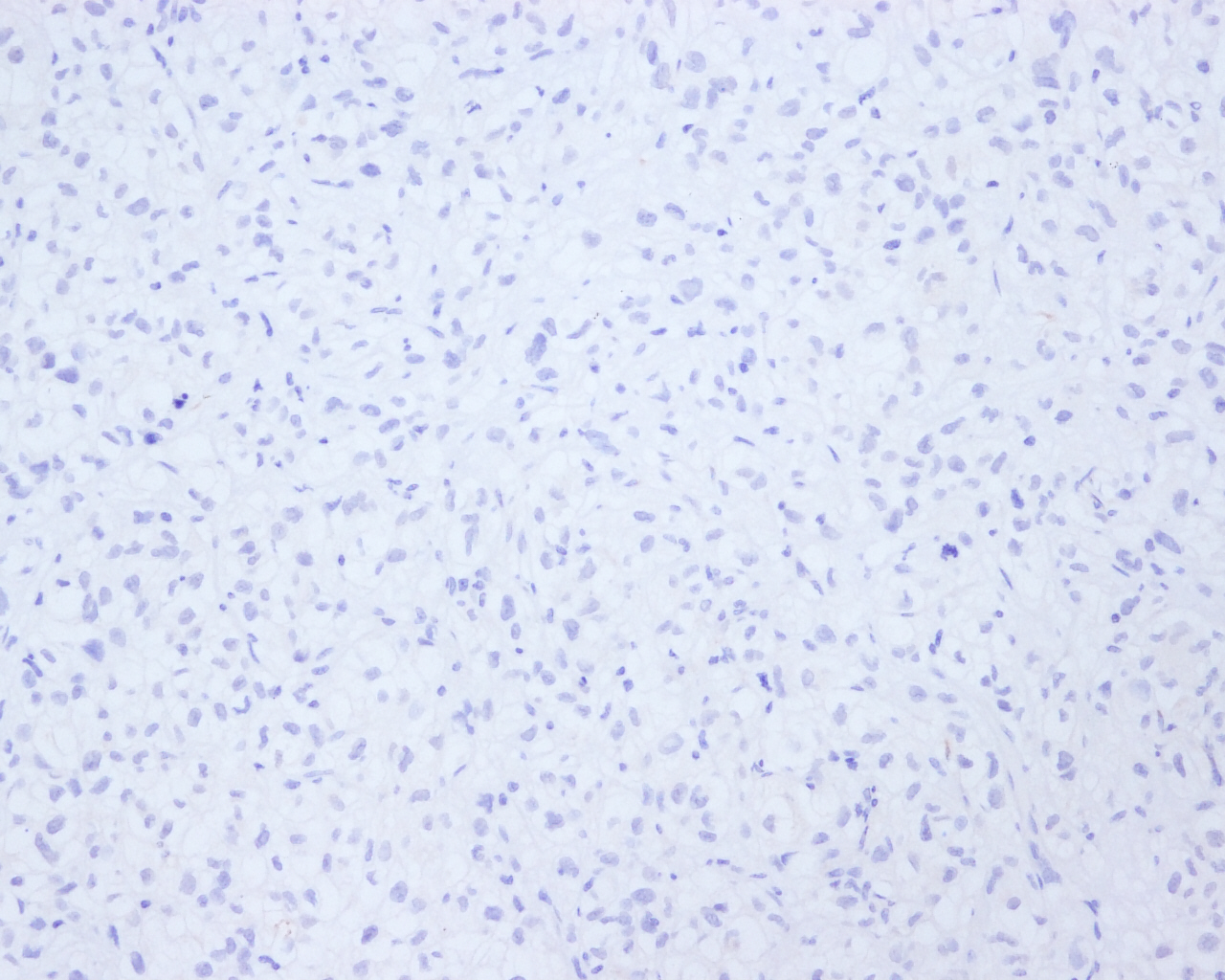

Supplement: Supplementary file 6 — Source Data for Figure 7 [file EMMM-12-e11889-s004.zip › EMM-11889-Fig7-source-data/Fig.7A_Ren28_inset.jpg]

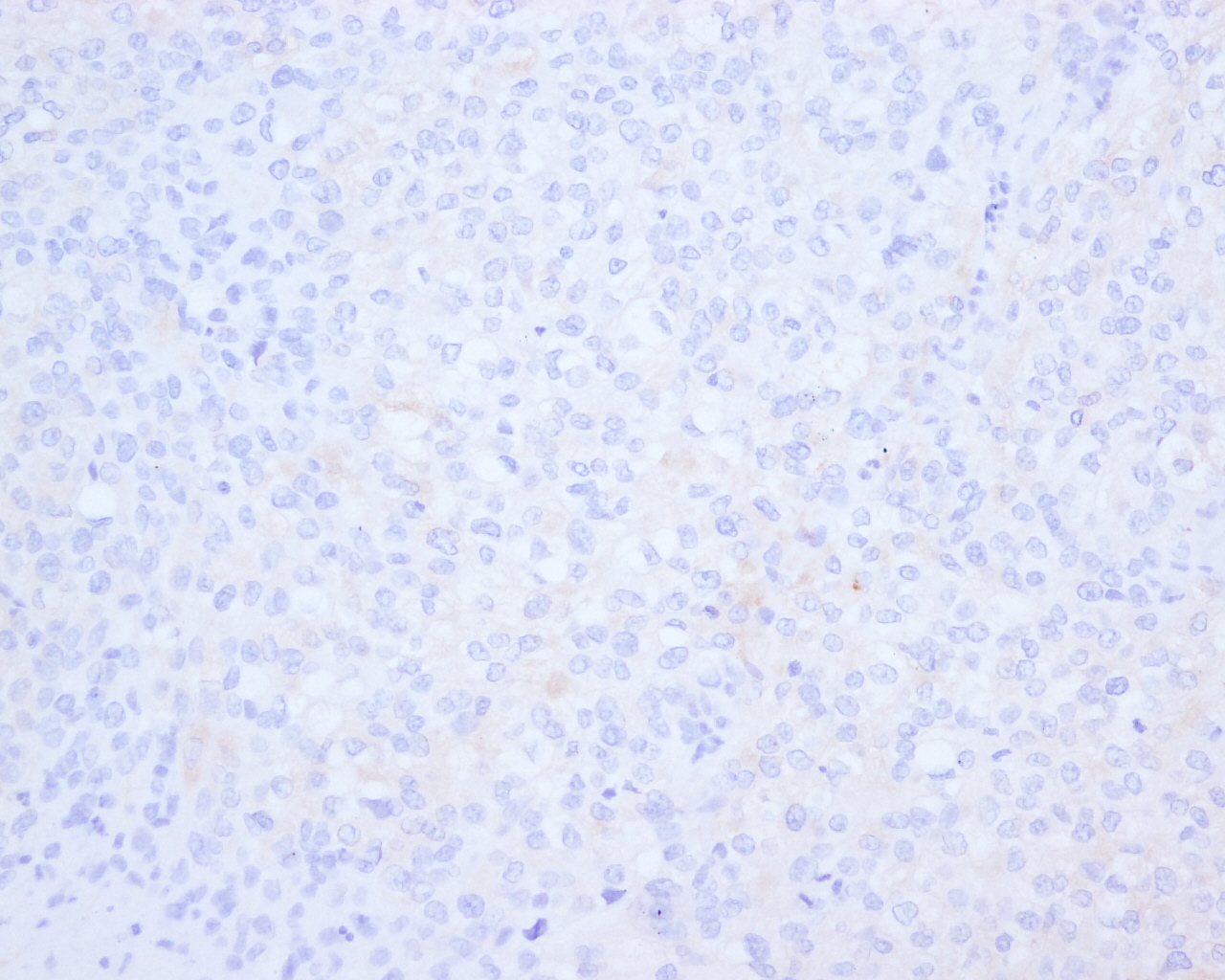

Supplement: Supplementary file 6 — Source Data for Figure 7 [file EMMM-12-e11889-s004.zip › EMM-11889-Fig7-source-data/Fig.7A_Ren50_inset.jpg]

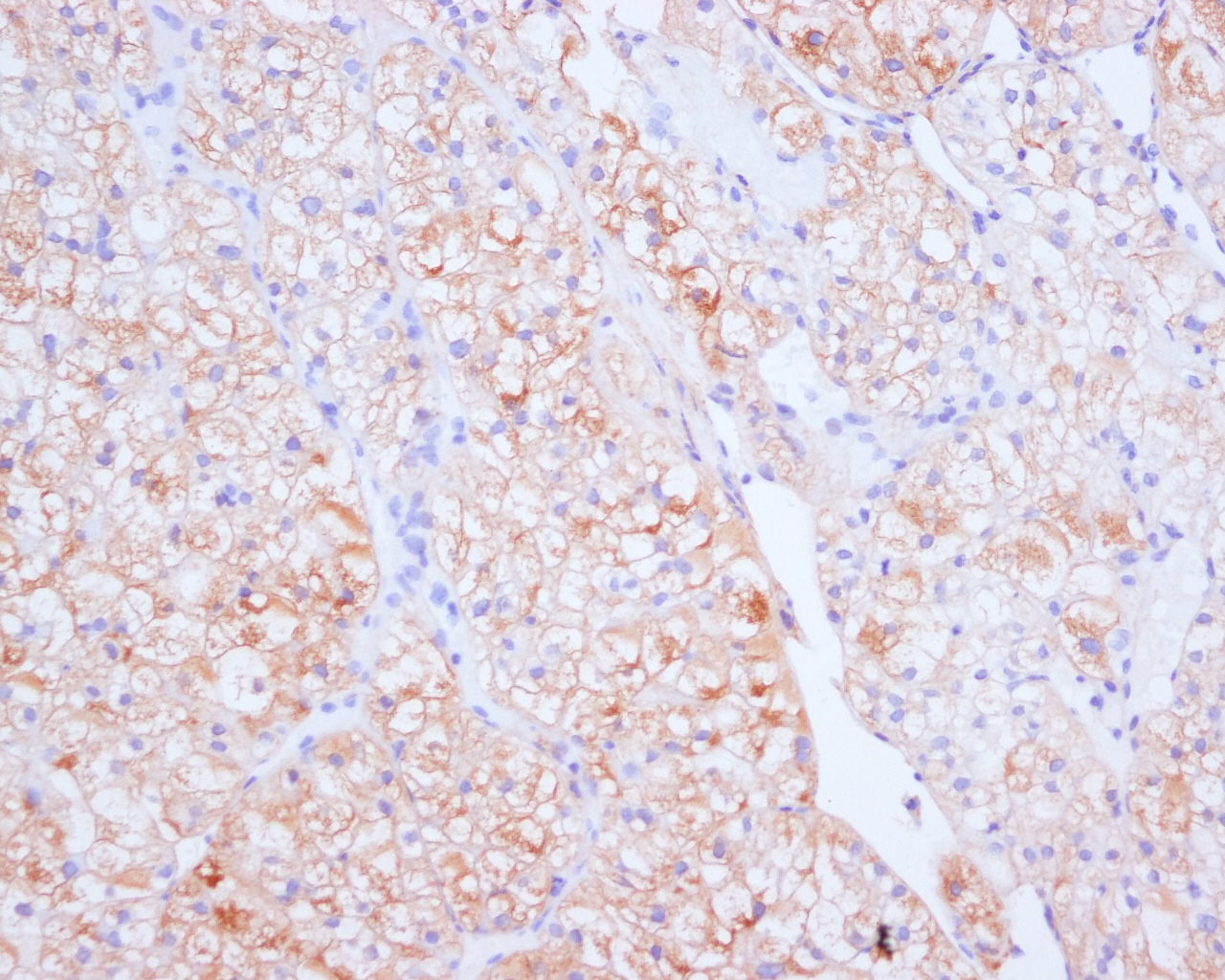

Supplement: Supplementary file 6 — Source Data for Figure 7 [file EMMM-12-e11889-s004.zip › EMM-11889-Fig7-source-data/Fig.7A_Ren86_inset.jpg]
